# How to properly treat endoscopic perforation

**DOI:** 10.1097/MD.0000000000047597

**Published:** 2026-02-06

**Authors:** Fengliang Dong, Jianwen Hu

**Affiliations:** a Department of Gastroenterology, Yiwu Tianxiang Medical Oriental Hospital, Yiwu, China; b Department of Gastroenterology, Dongyang People’s Hospital, Dongyang, China.

**Keywords:** abdominal pain, antibiotics, length of stay, perforation

## Abstract

This study aims to evaluate appropriate treatment measures after endoscopic closure of perforation. A retrospective analysis of patients undergoing endoscopic closure of gastric perforation was conducted to analyze whether stomach tube, fasting time, tumor site, antibiotics, operation time, and endoscopic method are related to body temperature and abdominal pain. Whether stomach tube, fasting time, antibiotics, tumor site, operation time, endoscopic method, body temperature, and abdominal pain are related to length of stay. Body temperature was correlated with fasting time and antibiotics, with statistical significance (*P* < .05), but not with gastric tube, lesion location, treatment method, and resection time; the incidence of abdominal pain was correlated with gastric tube, fasting time, and antibiotics, with statistical significance (*P* < .05), but not with lesion location, treatment method, and resection time. The length of stay was correlated with fasting time, body temperature, and abdominal pain, with statistical significance (*P* < .05), but not with gastric tube, lesion location, treatment method, antibiotics, and resection time. After perforation repair, it is feasible to use antibiotics according to the abdominal pain and body temperature of the patient. Gastric tube may cause abdominal pain and discomfort of the patient. Early oral feeding can shorten length of stay.

## 1. Introduction

With the progress of endoscopic treatment technology, endoscopic resection is widely used as one of the main therapeutic methods for gastric lesions because of its minimal invasiveness, high rate of en bloc resection, and comparable long-term outcomes to surgery.^[1–5]^ The incidence of endoscopic submucosal dissection (ESD) related perforation has been reported to be about 1.2% to 6.1%.^[6–8]^ In most cases, perforation can be successfully treated by immediate endoscopic closure and conventional treatment. Currently, numerous studies have reported on methods for endoscopic closure of perforation; however, few have addressed the observation and management of patients following closure. This study analyzes the relevant factors of abdominal pain, body temperature and hospital stay after endoscopic closure of perforation, and explores the appropriate treatment scheme after endoscopic closure.

## 2. Patients and methods

Patients who underwent endoscopic closure of gastric perforation at our hospital between October 2015 and January 2020 were retrospectively reviewed. Seventy-two endoscopic perforations were documented during the period. All endoscopic perforations were successfully closed using titanium clips. Informed consent was obtained from all patients prior to the endoscopic procedure. The data of stomach tube, fasting time, tumor site, antibiotics, operation time, endoscopic method, body temperature, abdominal pain, and length of stay were collected. Analyze whether stomach tube, fasting time, tumor site, antibiotics, operation time, and endoscopic method are related to body temperature and abdominal pain, and whether stomach tube, fasting time, antibiotics, tumor site, operation time, endoscopic method, body temperature, and abdominal pain are related to length of stay.

The Ethics Committee of Dongyang People’s Hospital approved this study. All participants signed an informed consent form.

### 2.1. Endoscopic treatment procedure

ESD and endoscopic submucosal excavation (ESE) procedures were performed by 2 endoscopists. An endoscopy produced by Olympus was used in this procedure. A transparent cap will be attached to the tip of the endoscopy. Carbon dioxide was used for insufflation. After intravenous anesthesia with propofol, Marks were made around the gastric lesion using a mucosal incision knife. Then, a submucosal injection of mixed solution of normal saline and methylene blue was administered. An IT-knife 2, a hook knife or a dure knife produced by Olympus was used to incise the superficial mucosa along the outer edge of the marker point and peel off the submucosa and lesions by endoscopists, and a snare was used to removal the lesion if necessary. If the gastric lesion arises from the submucosal or muscularis propria layer, ESE was used. On the bases of ESD technology, continue to gradually peel off the submucosa and part of the muscularis propria at the base of the tumor. Hemostatic forceps produced by Olympus or argon plasma coagulation were used for intraoperative hemostasis. The endoscopic mucosal resection procedure was as follows: Aspirating the tumor into a transparent cap (Saeed multi-band ligator, MBL-6-F; Cook), releasing the band, and cutting the ligated tissue with a snare. Aitanium clips produced by Olympus were used to close the perforation. After the endoscopic procedure, the physician empirically placed a nasogastric tube.

### 2.2. Postoperative management

The patients were transferred back to the ward for fasting and observation after recovery from anesthesia. All patients received fluid replacement and acid-suppressive therapy. Intravenous antibiotics or nasogastric tubes were used according to the doctor’s experience. The nasogastric tube was removed when the patient had no abdominal pain or bleeding. Patients in the ward had their body temperature measured every 2 hours and resumed oral intake after the abdominal pain resolved.

### 2.3. Statistical analysis

Baseline characteristics of patients and the length of stay are expressed as x±s means (standard deviation). The Pearson correlation coefficient was employed for correlation analysis, with a *P*-value .05 considered statistically significant. All statistical analyses were conducted using SPSS version 21.0 software (IBM Corporation, Armonk).

## 3. Results

### 3.1. Cohort characteristics

1. There were 72 patients with perforation, with an average age of 58.5 ± 11.0 years, including 47 females and 25 males; 31 cases were treated with antibiotics after endoscopic closure of perforation. 14 cases had fever >38°C, 39 cases had abdominal pain, 32 cases had gastric tube, the average endoscopic treatment time was 53.8 ± 33.0 minutes, the average length of stay was 11.2 ± 3.0 days, and the average fasting time was 2.9 ± 1.3 days. Forty-five patients with gastrointestinal stromal tumors have perforation. Forty-five patients fasted for 2 days. All perforations were managed successfully, and no mortality was reported.

### 3.2. Correlation analysis

1. Body temperature was correlated with fasting time and antibiotics, with statistical significance (*P* < .05), but not with gastric tube, lesion location, treatment method, and resection time; the incidence of abdominal pain was correlated with gastric tube, fasting time and antibiotics, with statistical significance (*P* < .05), but not with lesion location, treatment method and resection time, shown (Table 1).
2. The length of stay was correlated with fasting time, body temperature, and abdominal pain, with statistical significance (*P* < .05), but not with gastric tube, lesion location, treatment method, antibiotics, and resection time, shown (Table 2).

**Table 1:** Correlation between symptoms and treatment measures.

		Body temperature	Abdominal pain
Gastric tube	CC*	0.180	0.291**
	*P*	.131	.013
Fasting time	CC	0.304***	0.459***
	*P*	.009	.000
Lesion location	CC	0.171	0.128
	*P*	.150	.286
Antibiotic use	CC	0.308***	0.371***
	*P*	.009	.001
Treatment method	CC	‐0.061	0.067
	*P*	.610	.577
Resection time	CC	0.138	0.228
	*P*	.249	.054

* Correlation coefficient.

** *P* < .05.

*** *P* < .01.

**Table 2:** Correlation between length of stay and treatment measures.

		Length of stay
Gastric tube	CC*	0.108
	*P*	.368
Fasting time	CC	0.388**
	*P*	.001
Lesion location	CC	0.135
	*P*	.258
Antibiotic use	CC	0.168
	*P*	.158
Treatment method	CC	‐0.119
	*P*	.321
Resection time	CC	0.143
	*P*	.230
Body temperature	CC	0.270***
	*P*	.022
Abdominal pain	CC	0.301***
	*P*	.010

* Correlation coefficient.

** *P* < .01.

*** *P* < .05.

## 4. Discussion

With advances in diagnostic and therapeutic gastrointestinal endoscopy, many crucial endoscopic techniques are employed in clinical practice, such as endoscopic mucosal resection, ESD, and ESE. The incidence of perforation is increasing owing to procedural difficulties associated with endoscopy, anatomical sites of perforations, and endoscopists’ experience.^[9,10]^ Surgical intervention is the conventional treatment for gastrointestinal perforations. However, in recent years, with advancements in endoscopic equipment and techniques, numerous endoscopic devices are now utilized in the treatment of endoscopic perforations, such as endoscopic clips, over-the-scope clips, and stenting.^[11]^ Conservative management of gastrointestinal perforation includes gastrointestinal decompression, fasting, intravenous nutritional support, and administration of intravenous antibiotics.^[12]^ A previous study reported that antibiotics may be unnecessary for fever in patients without perforation and without serious comorbidities after gastric endoscopic treatment.^[13]^ In case of perforation during endoscopic treatment, endoscopic repair will be taken immediately to prevent leakage of cavity contents. At present, a large number of literatures have reported the endoscopic perforation repair technology, and lack of focus on post-perforation-repair management. Fasting, rehydration, gastric tube insertion and intravenous antibiotics became the basic treatment measures. However, whether these treatment measures are necessary is not mentioned in the literature. With the popularization of the concept of rapid rehabilitation and comfortable medical care, this study explores whether these treatment measures are necessary.

We found that antibiotics was significantly related to abdominal pain and body temperature, and it was effective for patients with abdominal pain and fever after repair of perforation. In this study, according to the doctor’s experience, the doctor will generally use antibiotics when the patient has fever or abdominal pain. For asymptomatic patients after repair of perforation, antibiotics may not be used temporarily. The mechanism of post-endoscopic procedural fever remains unclear. Previous studies have indicated that post-endoscopic procedural fever may be related to wound exposure and bacteremia.^[14]^ The etiology of abdominal pain may involve chemical stimulation by gastric juice, bacterial infection, or wound exposure. Currently, these factors cannot be clearly distinguished. Timely diagnosis of gastrointestinal perforation is critically important, because the leakage of gastrointestinal contents into the peritoneal cavity may induce fatal infection. Fasting before endoscopic treatment emptied the stomach, the gastroscope sucked up the gastric juice, moreover, perforations during endoscopic treatment were usually clipped quickly to prevent gastric juice from flowing into the abdominal cavity. These measures reduce the chance of abdominal infection. Many studies have shown that bacteremia, exposure of large wounds and long operation times may be correlated with pyrexia in patients treated after ESD or ESE,^[15–17]^ although continuous submucosal defects caused by ESD or ESE may increase the risk of bacteremia and/or endotoxemia, the rate of bacteremia remains low or the bacteremia is transient. Therefore, prophylactic antibiotics were not required for patients following endoscopic procedures.^[14,16–19]^ Inappropriate use of antibiotics will increase the cost and may cause adverse reactions, such as allergies, drug resistance and secondary infection.^[20–22]^ It is more beneficial for patients to use antibiotics as needed. In our study, among the patients who tested white blood cell, C-reactive protein and procalcitonin, some patients with abdominal pain and fever did not have elevated leukocytes, C-reactive protein and procalcitonin, some patients had elevated leukocytes and C-reactive protein but not procalcitonin, and some patients had elevated leukocytes, C-reactive protein and procalcitonin. It may be more objective to use antibiotics according to the white blood cells than according to the doctor’s experience.

In this study, the fasting time was related to abdominal pain and body temperature, mainly because the diet was generally opened when the abdominal pain was relieved. In this study, the average open time of diet was 2.9 ± 1.3 day. Generally, the open time was 2 days after fasting. Oral feeding and enteral nutrition as early as possible are helpful to the early recovery of patients. As long as the perforation is fully clipped, whether the patients with abdominal pain can also open the diet early needs further research. For patients with perforation, gastrointestinal decompression is one of the basic treatment measures. This study found that gastric tube increased abdominal pain, which was not related to body temperature and did not reduce the discomfort symptoms of the patients. Gastric tube is not necessary after endoscopic perforation is fully clipped. In this study, the endoscopic treatment method, tumor location and treatment time related to the symptoms. Although the treatment time is prolonged, once the perforation occurs during the operation. The endoscopist will clip the perforation immediately.

Patients with endoscopic perforations require antibiotic therapy and fasting observation, leading to prolonged hospitalization.^[12]^ We found that the length of stay was related to fasting time, so early open diet was helpful to the early recovery and discharge of patients. The length of stay was related to fever and abdominal pain and not related to intravenous antibiotics. We speculate that the possible causes of these fever and abdominal pain were mainly caused by chemical stimulation, endoscopic injury or wound exposure.

Limitations of this study are as follows: This study was designed as a single-center investigation, and its conclusions require further validation through multicenter studies. All endoscopic procedures were performed by 2 endoscopists. Given the variability in operators’ experience, future research should incorporate a broader range of practitioners to enhance the generalizability of the findings. Decisions regarding antibiotic use and gastrointestinal decompression were based on the empirical judgment of clinicians, which may introduce selection bias.

With the progress of endoscopic treatment technology, technology of endoscopic perforation repair has been mature. After perforation repair, it is feasible to use antibiotics according to the abdominal pain and body temperature of the patient. Using antibiotics as needed can effectively control infection. Gastric tube may cause abdominal pain and discomfort of the patient. After the endoscopic perforation is clipped, there is no need for routine gastric tube insertion. Early oral feeding can shorten length of stay. The value of anti-infection based on blood routine, C-reactive protein, procalcitonin, and other indicators needs further study.

## Author contributions

**Conceptualization:** Fengliang Dong.

**Investigation:** Fengliang Dong, Jianwen Hu.

**Writing – review & editing:** Jianwen Hu.
